# Revitalizing physical assessment in undergraduate nursing education - what skills are important to learn, and how are these skills applied during clinical rotation? A cohort study

**DOI:** 10.1186/s12912-019-0364-9

**Published:** 2019-09-05

**Authors:** H. Ösp Egilsdottir, Kirsten Røland Byermoen, Anne Moen, Hilde Eide

**Affiliations:** 1 Science Centre Health and Technology, Faculty of Health and Social Sciences, University of South-Eastern Norway, Grønland 58, 3045 Drammen, Norway; 2 Institute for Health and Society, Faculty of Medicine, University of Oslo, Kirkeveien 166, Fredrik Holsts hus, 0450 Oslo, Norway

**Keywords:** Physical assessment skill, Nursing students, Nursing education, Health assessment

## Abstract

**Background:**

The preparedness of newly graduated registered nurses for a demanding work environment and care practices takes form during nursing education. Norwegian nursing education at one university has implemented a selection of basic physical assessment skills (B-PAS) in the nursing curriculum in order to prepare nursing students for a demanding work environment post-graduation.

**Methods:**

A mixed-method cohort design. We evaluated nursing students’ self-reported use of B-PAS during their clinical rotation using the “Survey of Examination Techniques Performed by Nurses” questionnaire (30 items). In addition, two focus group interviews elicited factors that hinder or facilitate the actual use of B-PAS during clinical rotation. We recruited students from a bachelor’s degree programme for nursing at a Norwegian university. Three hundred and sixty-three of 453 eligible nursing students in the first, second, and third year of the bachelor’s degree programme participated in the study (80%).

**Results:**

ANOVA showed a significant progression (*p* < 0.016) in students’ self-reported use of B-PAS. Auscultation and percussion skills were graded below the median score of 3, which indicates that these skills were less used throughout the programme. The nursing students highlighted contextual factors for their use of B-PAS when in clinical rotation. Preceptors are important gatekeepers for successful implementation, and there is a need for close collaboration between the university and clinical practice.

**Conclusion:**

Despite the reduced PAS taught in the curricula, there is still a lack of application of such skills in clinical rotations. This study highlights that research should explore how different work environments influence the utilisation of learned skills, and which learning strategies are appropriate or most successful for stimulating clinical reasoning and the extensive use of physical assessment.

## Background

A rapidly changing work environment puts novice registered nurses’ (RNs) abilities for clinical reasoning and clinical judgment to the test in all contexts. Adequate patient assessment in different clinical encounters can be challenging for newly graduated RNs because of increasingly complex healthcare needs, chronic disease, comorbidity, and polypharmacy [11, 20]. Novice RNs must master the ability to make decisions based on solid general health assessments and physical assessments; for example, by determining what data are important to collect and then choosing the right interventions in the correct order [15]. Several studies indicate that inexperienced RNs struggle to process large amounts of complex data, to anticipate changes in a patient’s situation, and to differentiate between clinical situations that need immediate attention and those that are less acute [15, 16, 22]. This can threaten patient safety and could result in near misses and adverse patient outcomes [16, 22].

There is a general agreement that health assessment and physical assessment are core competencies within the scope of nursing practice [3, 19, 30, 32, 33]. The RNs’ preparedness for demanding work environments and care practices takes form during the nursing degree programmes. Research has shown that it is important to articulate knowledge from human bioscience (anatomy, physiology, pathology, pathophysiology, and pharmacology) when assessing and interpreting the collected data [4, 18]. A persisting challenge is how the pedagogical methods and curriculum in nursing education influence and prepare nursing students for demanding clinical situations, especially regarding patient assessment and clinical reasoning [16].

Internationally, general health assessment and physical assessment are well-integrated in undergraduate nursing curricula, for example, in the US since the 1970s and in Canada, New Zealand, and Australia since the 1990s [1, 3, 5, 14, 17, 21]. However, a literature review and newer studies focusing on physical assessment in undergraduate nursing curricula show that newly graduated RNs are not using all of the physical assessment skills (PAS) they were taught in their clinical practice [3, 9, 21]. The RNs only use a subset of acquired PAS [2, 3, 21, 30]. This situation is not new and has been debated since Giddens [13] published her survey results focusing on the PAS that RNs use in their work. The highlights of the debate suggest that a critical review of the range of PAS taught in nursing education is necessary because these skills do not always reflect the RNs’ or the nursing students’ scope of practice [3, 9, 11, 13, 31]. More recent studies suggest, rather than reducing the number of PAS being taught, ensuring that the education programmes allow sufficient time on campus for the application and interpretation of patient assessment skills before and during the clinical rotation [3, 9, 33]. Perceived barriers for routinely adapting and implementing physical assessment in RNs’ daily practice include doubts about the total impact on the patient outcome, a lack of confidence in performing physical assessments, and a lack of role models within the nursing profession [9].

### Physical assessment in Norwegian nursing education

In Norway, the role of the nurse practitioner (NP) has recently been introduced in clinical practice, including the use of PAS. Following the implementation of PAS at the master’s level, physical assessment was first documented in a bachelor’s level curriculum in 2013 [4]. This means that there are few role models in clinical practice, and possibly little competent guidance for nursing students to apply these skills in direct patient care.

Fifty percent of Norwegian nursing education takes place in clinical rotation in different contexts [24]. In our University, seven clinical rotation periods are spread over three years. The nursing students are assigned preceptors in all clinical rotations and are supported by faculty. During the first year, the 8-week clinical rotation takes place in primary healthcare, in either home care or nursing homes. During the second year, the students have three clinical rotations – two at a hospital in the medical and surgical wards (8 weeks), and one related to health promotion (2 weeks). During the third year, the students are back in primary healthcare. One rotation takes place in home care, one rotation in nursing home care, and one in mental health care (all 8 weeks each).

In 2015, a carefully selected range of PAS was implemented in the curriculum in undergraduate nursing at our University. These included:

- the heart and peripheral circulatory system (11 skills)
- the respiratory system (7 skills)
- the abdominal system (6 skills)
- the neurological system (6 skills)

These PAS were considered to be the basic competence for bachelor’s degree students, and are referred to as *basic* PAS (B-PAS) in the nursing curriculum at the university. There were three main reasons for the selection of these skills. First, other studies have shown that there is limited use of PAS in clinical practice [3, 9, 13, 30]. Second, the existing nursing curriculum was already saturated, and it was not possible to remove elements to make room for the full range of PAS. Third, the newly started master’s programme for NPs also included a physical assessment course with a broader focus. Therefore, it was important to establish an understanding of the differences in scope of practice between the RN level and the NP level.

**Fig. 1:**
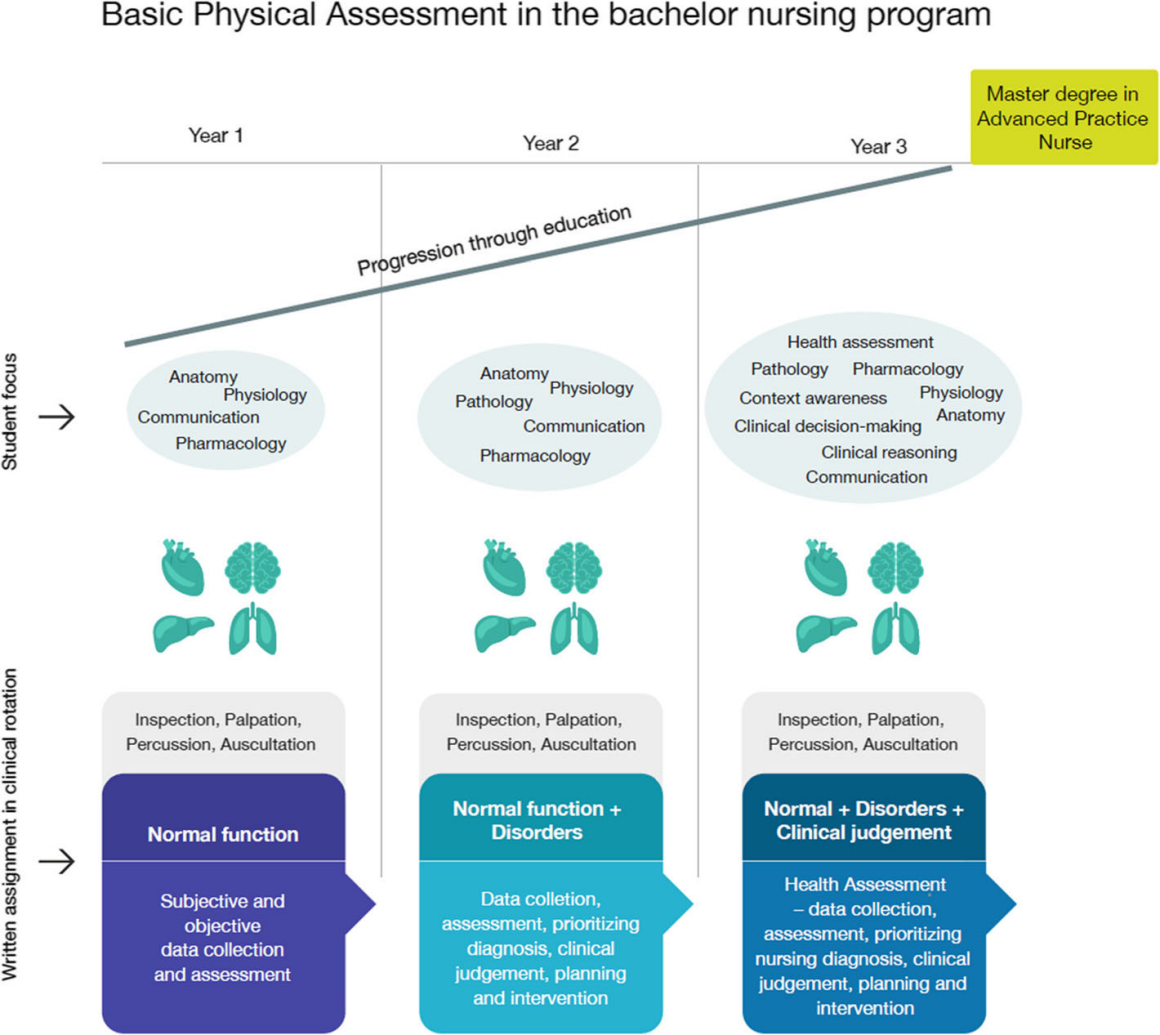
Progression model

We developed a progression model to scaffold students’ B-PAS development throughout their nursing education (Fig. 1).

Figure 1 illustrates the relationship between and the progression in developing B-PAS and educational courses in clinical rotation throughout the nursing programme.

## Method

The aim of this study was to evaluate nursing students’ self-reported use of B-PAS in clinical rotation after implementation of this component in their nursing education. We also sought to identify factors that inhibit or encourage the nursing students in the use of B-PAS and how these factors can influence the students’ development of competence and confidence in applying these skills. The study had a mixed-method cohort design, collecting data using self-reporting survey questionnaires and focus group interviews. The focus group interviews enabled the researchers to focus on specific elements in the questionnaire. By using mixed methods, the results from the different data sources can complement and strengthen each other [28, 29].

### Data collection

During spring 2017, all first-year students (cohort 2016) and second-year students (cohort 2015) at our campus were invited to participate in the survey and in one focus group interview. The third-year students that year were not included in the current study because they were only introduced to B-PAS during the last year of their studies. The included students had all been taught B-PAS from their first year of study. Cohort 2015 was also invited to answer the questionnaire at the end of their third year, during spring 2018.

All of the students had finished their clinical rotation in the educational year in which the data collection was carried out. An invitation with information was published on the University’s web-based learning platform. The researchers then met the students to provide additional information about the study when handing out a paper-based questionnaire after lectures on campus. Participants in the focus group interviews volunteered directly to the researchers, at which time additional information was provided. Table 1 gives an overview of the design, student cohorts, data collection time, and measures.

**Table 1:** Overview of design, student cohorts and data collection time

	Spring 2017		Spring 2018
	Questionnaire	Focus group interview	Questionnaire
Student cohort 2015 Started autumn 2015 in the nursing programme	x	x	x
Student cohort 2016 Started autumn 2016 in nursing programme	x	x	

Use of B-PAS was surveyed with 30 items selected from the “Survey of Examination Techniques Performed by Nurses” developed by Jean Giddens in 2007. The original validated instrument contains 122 physical assessment items. Modification of the original instrument was necessary due to the focus on B-PAS, and our questionnaire measures the taught skills.

The questions asked about the extent to which the students use the different elements in physical assessment with a Likert scale of six possible answer categories: (1) *I do not know how to do this technique*, (2) *I know how to do this technique, but have never done this in my clinical practice*, (3) *I perform this technique rarely*, (4) *I perform this technique occasionally*, (5) *I perform this technique frequently in my clinical practice*, and (6) *I perform this technique regularly in my clinical practice*. Participants were asked to rate the frequency with which they performed each skill in the clinical setting. Approval to use and restructure the questionnaire was given by Giddens in January 2017.

Five background variables were also collected, including year of nursing school, gender, age, healthcare-related work or other work-related experiences prior to entering nursing education, and the number of paid working hours while attending nursing school.

From our experience as faculty working with nursing students in clinical rotation and the collaboration between nursing students and preceptors, elements in the Norwegian context were identified that could influence the use of B-PAS in clinical rotation. The students were asked: “Which of following factors will increase the use of basic physical assessment skills in clinical rotation?” with the following six possible answers: (1) *Collaboration with fellow students*, (2) *More collaboration with the preceptor during clinical rotation*, (3) *More facilitation from faculty*, (4) *Instruction videos*, (5) *An online course*, or (6) *None of the mentioned choices*. The students marked the factors they agreed would support the use of B-PAS. They could mark as many factors as they wished.

To acquire a deeper understanding of the results from the questionnaire, two focus group interviews were conducted after the students answered the questionnaire, one with first-year nursing students (cohort 2016) (*n* = 6, duration 45 min) and one with second-year students (cohort 2015) (*n* = 7, duration 55 min). The interviews were semi-structured with open-ended questions. The questions focused on a) the general use of B-PAS, b) the contextual factors that encouraged or hindered these assessment skills being used in clinical rotation, and c) the factors that would increase the learning outcome when developing and applying the skills. The nursing students spoke freely about their own experiences and reflected on their time in clinical rotation. The researchers stimulated the students to talk together without influencing their reflections [10]. During the interviews, the researchers used follow-up questions if there were unclear statements that required elaboration.

### Research ethics

The Norwegian Centre for Research Data (NSD) approved the study (Project No. 53525). All students were informed about confidentiality and voluntary participation. The study information and invitations to participate in the study were introduced in writing through the school’s learning management platform. Written information included the aim of the study and how data would be collected by using self-reported questionnaires and focus group interviews. The researchers also gave oral information in the classroom when there was an opportunity to do so.

### Data analysis

Descriptive statistics were used to analyse demographic and B-PAS data in the questionnaires (SPSS 24 Inc., 2017). The B-PAS data used a Likert scale for calculation of mean and frequency and median scores to measure central tendency. To allow comparison with similar studies [3, 13, 19], the mean and standard deviation are presented for more insight and understanding. One-way between-group analysis of variance of independent groups (ANOVA) was conducted to explore the development in the use of B-PAS between all three cohorts as follows: development between the first and second year, between the second and third year, and between the first and third year. Due to multiple comparisons, the *p* value was set to 0.016 using Bonferroni correction. We also calculated effect sizes (Cohen’s *d*) to evaluate degree of change, which was categorized as small (< 0.5), medium (0.5–0.8), or large (> 0.8) [6].

The focus group interviews were analysed using content analysis, drawing from Elo and Kyngäs [10]. The interviews were transcribed verbatim and read thoroughly to acquire an overview of how the students elaborated on their experiences in clinical rotation [10]. Combing our focus on B-PAS and students’ experiences, central themes were identified, further abstracted as subcategories, and then presented as main categories [10].

## Results

The results of the questionnaire highlight the B-PAS used in clinical rotation by the nursing students, as well the progression between the educational years, while the results from the focus group interviews highlighted the contextual factors in the work environment in the clinical rotations.

Of the 453 students who were invited to participate, 363 returned the survey questionnaire fully completed (an overall response rate of 80%). By class cohort, the response rates were as follows: first year (cohort 2016), 146 of 184 (79.4%); second year (cohort 2015), 127 of 142 (89.4%); and third year (cohort 2015), 90 of 127 (70.9%).

Consistent with the university’s overall demographic data profile, the sample consisted primarily of women (84.6%, *n* = 307) with a mean age of 25.3 years. About half (50.4%, *n* = 183) had no experience in healthcare before starting the Bachelor’s programme, 24.2% (*n* = 88) had 1–2 years of experience, 16.8% (*n* = 61) had 3–5 years of experience, and 8.6% (*n* = 31) had more than 6 years of experience. The majority of the students (56.2%, *n* = 205) worked about 1 to 2 shifts weekly (7.5 to 15 h) during nursing school, while 29.2% (*n* = 106) did not work any extra hours in healthcare services during nursing school.

The focus groups consisted of 13 participants – six men and seven women – ranging in age from 23 to 49 years.

### Survey findings

The students self-reported their knowledge and use of the 30 different PAS on a Likert scale of 1–6. Seven skills had a median score of 4 or higher in all cohorts, indicating that the students used the skills “occasionally to regularly” during clinical rotation. Altogether 13 skills were scored with a median score of 4 or higher throughout all three years. Thirteen skills had a median score of 3 for all cohorts, which indicates rarely using these skills.

ANOVA of the mean scores showed that all respondents knew how to perform the different types of physical assessment. However, Table 2 shows that there was some variation in how often students reported using the skills. The main progression in use of B-PAS was between the first and third year, and Table 2 shows that the mean difference was statistically significant at *p* < 0.016 in 19 of the 30 implemented skills. The actual difference in mean and median scores between the groups was small; however, there was a change and development in the student’s use of B-PAS as indicated by Cohen’s *d* with 17 of the total 30 skills having medium to large effect sizes. No demographic data reached statistical significance in correlation analysis of the usage of B-PAS.

**Table 2:** Development of basic physical assessment skills over the 3-year nursing programme

Physical assessment skills	Mean (*SD*), Median			Cohen’s *d* effect size	
	1st year	2nd year	3rd year	1–2 year	1–3 year
Heart and peripheral circulation					
Inspect extremities for skin colour/hair growth	4.51 (1.17), 5	5.13 (3.69), 5	4.73 (1.05), 5	0.22	0.19
Palpate distal pulses	3.62 (0.96), 4	4.28 (1.12), 4	4.22 (1.07), 4	0.63*	0.59*
Count pulses ^a,b^	4.23 (2.57), 4	5.14 (0.80), 5	4.99 (0.87), 5	0.48	0.39
Palpate for oedema ^a,b^	3.65 (1.16), 4	4.20 (1.06), 4	4.38 (0.99), 4	0.50*	0.67*
Palpate and inspect capillary refill ^a,b^	2.78 (1.21), 3	3.76 (1.21), 4	3.77 (1.11), 4	0.80*	0.85*
Estimate skin fold ^a,b^	2.86 (0.94), 3	3.30 (1.08), 3	3.56 (0.91), 4	0.43	0.75*
Evaluate extremities for skin sensation ^a,b^	2.30 (1.10), 2	3.10 (1.15), 3	2.92 (0.87), 3	0.71*	0.62*
Assess fine motor skills	2.62 (1.27), 2	2.98 (1.23), 3	2.98 (0.96), 3	0.28	0.31
Take blood pressure ^b,c^	3.91 (1.04), 4	3.61 (1.09), 4	4.29 (1.09), 4	-0.28	0.35
Auscultate heart sounds	2.52 (1.02), 2	2.32 (1.01), 2	2.57 (0.88), 2	-0.19	0.05
Auscultate carotid artery	2.60 (0.91), 2	2.52 (1.16), 2	2.76 (0.97), 3	-0.07	0.17
Thorax					
Inspect thorax for shape, breathing effort ^a,b^	3.55 (1.16), 4	4.45 (1.25), 5	4.31 (1.06), 4	0.74*	0.68*
Count respiratory rate ^a,b,c^	3.99 (0.91), 3	5.57 (0.65), 6	5.09 (0.87), 5	1.99*	1.23*
Inspect thorax for skin colour/scar ^a,b^	4.28 (1.22), 4	5.13 (0.83), 5	5.97 (0.77), 5	0.83*	1.63*
Palpate thorax wall for thoracic expansion and vocal fremitus ^a,b^	2.82 (1.03), 3	3.41 (1.34), 3	3.27 (1.07), 3	0.50*	0.42
Percuss the lungs ^b^	2.41 (0.79), 2	2.52 (1.02), 2	2.87 (0.93), 3	0.12	0.53*
Auscultate lungs	2.70 (0.86), 3	2.76 (1.02), 3	3.13 (0.99), 3	0.06	0.46
Assess SpO_2_^a,b^	3.83 (1.31), 4	5.63 (0.66), 6	5.03 (1.02), 5	1.73*	1.02*
Abdomen					
Inspect abdomen ^a,b^	2.95 (1.21), 3	3.87 (1.06), 4	3.74 (1.07), 4	0.80*	0.69*
Auscultate abdomen for bowel sounds ^b^	2.61 (0.86), 2,5	2.90 (1.06), 3	3.02 (0.92), 3	0.30	0.46
Light abdominal palpation ^a,b^	2.62 (0.89), 3	3.13 (1.05), 3	3.36 (0.90), 3	0.52*	0.82*
Deep abdominal palpation	2.21 (0.78), 2	2.65 (1.00), 2	2.70 (0.90), 3	0.49	0.58*
Percuss the abdomen ^b^	2.36 (0.79), 2	2.52 (0.99), 2	2.77 (0.81), 3	0.17	0.51*
Percuss for kidney tenderness ^a,b^	2.05 (0.84), 2	2.41 (0.97), 2	2.57 (0.81), 2	0.39	0.63*
Neurology					
Evaluate CN I-XII	1.82 (0.88), 2	1.88 (1.73), 1	2.20 (1.09), 2	0.04	0.38
Evaluate muscle strength, atrophy, tone	2.63 (1.13), 2	2.73 (1.26), 3	2.79 (1.06), 3	0.08	0.14
Evaluate sensation of touch ^a,b^	2.28 (1.12), 2	2.89 (1.2), 3	2.96 (0.91), 3	0.52*	0.66*
Assess coordination and balance	3.16 (1.13), 3	3.43 (1.46), 3	3.32 (1.23), 3	0.20	0.13
Evaluate patella and plantar reflexes	2.25 (0.67), 2	2.36 (0.77), 2	2.47 (0.80), 2	0.15	0.29

^a^mean difference is significant at the 0.016 level between 1^st^ - 2^nd^ year

^b^mean difference is significant at the 0.016 level between 1^st^ - 3^rd year^

^c^mean difference is significant at the 0.016 level between 2^nd^ - 3^rd year^

^*^Cohen’s *d* effect size above 0.5

Assessments of neurology, percussion, and auscultation in abdominal, respiratory and circulatory systems had median scores of 3 and were thus seldom used. Table 3 illustrates the four skills that had a median score of 1 or 2.

**Table 3:** Basic physical assessment skills with median scores 1 and 2 in all cohorts

Auscultate heart sounds	
Percuss for kidney tenderness	
Evaluate CN I-XII	
Evaluate patella and plantar reflexes	

In the B-PAS for assessing the heart and peripheral circulatory system, six of eleven skills changed significantly. Four of the skills had a medium effect size, and one skill, palpate capillary refill, had a large effect size. In the B-PAS for assessing the respiratory system, six of seven skills changed significantly over the three years. Three skills – measuring the respiration rate, assessing SpO_2_, and inspecting the thorax – had a large effect size. Auscultation of the lungs did not reach statistical significance despite a medium effect in development over time. In the B-PAS for assessing the abdominal system, five of six skills had statistically significant development and, of these, four had a medium effect size and one, light abdominal palpation, had a large effect size. In the B-PAS for assessing the neurological system, two of five skills reached statistical significance, and the skill of evaluating sensation of touch had a medium effect and evaluating mental status had a large effect in use.

In the questionnaire, the nursing students were asked to indicate which factors might support increased use of B-PAS in their clinical rotation. The three most important factors were collaboration with fellow students, increased collaboration with preceptors in clinical rotation, and greater facilitation from faculty. Table 4 presents the students’ answers in prioritized order.

**Table 4:** What will increase the use of basic physical assessment skills in clinical rotation? (*N* = 363)

Cooperation with fellow students	67, 9% (*n* = 210)
More cooperation with nurse in clinical rotation	57, 3% (*n* = 208)
More facilitation from preceptor	42, 7% (*n* = 155)
Instruction videos	28, 9% (*n* = 105)
Online course	23,1% (*n* = 84)
None of the mentioned choices	5,2% (*n* = 19)

### Focus group findings

The focus group interviews broadened the understanding of the questionnaire results presented in Tables 2, 3 and 4. The interviews also gave insight into how nursing students experienced applying B-PAS skills and which challenges they met during clinical rotation. Table 5 presents the three main categories that were identified from the focus groups interviews.

**Table 5:** The main categories for the focus group interviews

1. Taking vital signs and being responsible for NEWS score are routine student assessments but doing more is challenging	
2. Skill development in clinical practice can be fostered by access to digital learning resources when in clinical rotation	
3. A culture for articulation of knowledge fundamental for clinical reasoning, clinical judgement, and self-efficacy in B-PAS	

Category 1 “Taking vital signs and being responsible for NEWS score are a routine student assessment, but doing more is challenging”: In the interviews, the nursing students talked about which tasks were quickly assigned to them at the beginning of the clinical rotations. Taking vital signs and being responsible for scoring the early warning score (EWS) (in Norway, National Early Warning Score, NEWS) was a routine student assignment in the clinical rotation. A second-year student said:

“*It’s like, especially related to NEWS-ing or assessing, the pulse, the respiration rate*”.

A first-year student also said:

“*It depends on what patients you meet, but there was a lot of checking for pulse and blood pressure and oxygen saturation*”.

At the same time, the students mentioned that because taking vital signs and “NEWS-ing” the patient data was so implemented as a student assignment, it provided a good opportunity to expand the assessment and use B-PAS to a certain extent:

*“Doing NEWS is very well implemented here* (in the hospital setting), *that you could combine these two* (the B-PAS and NEWS)*, let’s say that you are taking the blood pressure then you could also assess the peripheral circulation, like pulses and oedema right? If you combine these two* (the B-PAS and NEWS) *you all of a sudden have more information to make a better assessment (second-year student)”.*

The students also felt that opportunities to apply B-PAS in clinical rotation were dependent on the preceptor’s personal attitude towards the relevance of these new skills. If the preceptors welcomed B-PAS in their work methods, the students were encouraged to apply and practice the skills in direct patient care. However, if the preceptors expressed a negative attitude towards B-PAS, the students struggled to find opportunities to use these skills:

“*One of our preceptors had been attending a course here* (at the university) *and she was probably not a fan of B-PAS, so she thought it was, I don’t know, a little inconvenient that it took too much time, and that the first assessment was too extensive to make (second-year student)”.*

A first-year student highlighted this:

*“Luckily we were two students* (at the clinical rotation) *and it was really good to be two students. We were able to talk because we both knew what we were supposed be doing, and it was difficult to get an understanding about that from the staff on the unit, they didn’t stand in our way or ruin anything for us, but they* (the staff) *didn’t want to, didn’t understand what we were supposed to be doing there”*.

Some of the students also experienced that the preceptor commented directly that doing B-PAS was a physician’s work, not a nurse’s. This indicates that the students struggled to get support and encouragement to apply B-PAS in clinical rotation. Despite a lack of support, the students found it meaningful to try to apply some of the simpler B-PAS skills, like vital signs which are well-known nursing tasks.

Category 2 “Skill development in clinical practice can be fostered by access to digital learning resources when in clinical rotation”: In the nursing students’ opinion, it would have been helpful and supportive to have access to digital learning resources demonstrating B-PAS when in clinical rotation. This would give guidance related to questions or challenges that the students encountered and would support working with the basic physical assessments in clinical rotation. To quote one of the second-year students:

*“As a nursing student you really want to have the apps, because you feel supported if you can go in and get the information, it’s easier. If you have an app which was a collection of all the knowledge about these issues, with some instruction videos which you could watch and refresh the skills and B-PAS techniques, and then apply these in clinical rotation. Then it would be easier and more useable to apply it because you have doubts about whether what you are doing is according to standards”*.

In addition, the students wondered how available the knowledge related to B-PAS was for the preceptors; in terms of educating the preceptors and for the students to be able to show them what B-PAS was really about. However, the students valued the opportunity to practice with peer students and develop the skills at a university skill lab with faculty. One of the first-year students said:

“*It would have been nice, like at least before we were going into clinical rotation, if it had been possible to practice more. That is something I would have appreciated, because it’s just about having it* (B-PAS) *in your fingers*”.

Another first-year student followed up and stated that:

*“It would be good to practice now, after we have been in the clinical rotation and practiced properly, now we know more about what we need to practice on”.*

Still, the students also stated clearly that it would increase the learning outcomes in terms of skills development and proficiency to apply B-PAS in direct patient care. As one of the second-year students highlighted:

“*I’m thinking, it’s really good to work with a nurse or a physician, to inspect, palpate, practice, it was helpful that we did it in the simulation lab before clinical rotation, just to actually understand what this is about. But, it is different when you meet a sick patient and have to relate to what it is that you do, or what you are looking for. One thing is doing this on healthy persons* (fellow students) *but another is doing it on sick patients, when you have to try to understand why and how things are related”*.

It is clear that the nursing students’ learning experiences and opportunity to develop B-PAS depends both on being able to practice at the university with faculty as well as in the clinical context with preceptors.

Category 3 “A culture for articulation of knowledge fundamental for clinical reasoning, clinical judgement, and self-efficacy in B-PAS”: The nursing students also pointed out the need to focus constantly on the fundamental knowledge required to perform B-PAS. This fundamental knowledge includes anatomy and physiology, which explains the healthy body and body sounds. As well as pathophysiology and pathology, which explains the sick body. One of the second-year students highlighted this:

“*When you do not get a bit of it* (the knowledge in human science courses) *linked to practicing* (B-PAS), *I think it* (the knowledge in human science courses) *will be disconnected, and I have to try really hard to link these things* (the knowledge and the skills) *together on my own”.*

Another second-year student elaborated further:

“*It’s hard to get things to connect together because...maybe one should have anatomy and pathophysiology…a bit like continuously together for example with the heart, to learn about the healthy heart first and then learn about the sick heart”*.

Some of the students experienced valuable feedback from physicians which fosters the knowledge transition between fundamental knowledge and developing B-PAS. Both preceptors and physicians were regarded as important discussion partners on how to interpret the data collected via B-PAS. They supported discussions about the collected “cues” in the nursing assessment that promote clinical reasoning. The nursing students also emphasized that using B-PAS would contribute to better communication and collaboration between the nurses and physicians:

“*I had quite a lot of collaboration with the doctor and I think that the doctor assumed that I was a third-year student because she used very many medical terms (first-year student)*”.

A “safe” environment was necessary to feel comfortable enough to ask questions and to discuss observations with both nurses and physicians after performing B-PAS, especially regarding the meaning and interpretation of possible findings. One of the second-year students said:

“*We made our own assessments. Then I went back to the doctor and my preceptor to discuss what we had seen, and in many cases, it matched. Then you get that feeling that this is something you can master and that you can become a good nurse*”.

Further emphasizing the importance of good communication, one second-year student said:

“*To have someone to discuss things with, who does not think that you are asking stupid questions. That this is useful learning and that we sit here and discuss it, that you dare to test out these skills and that you dare to say what you think, that you can accept receiving feedback for how you can become better and such things. That is how you become more confident*”.

These statements indicate that the cultural factors in the work environment influence students’ learning and knowledge transitions. Thus, good communication and collaboration between nurses and physicians in the clinical rotation units are essential for the nursing student to develop clinical reasoning and develop B-PAS.

## Discussion

The current study reports the findings from the first evaluation of the implementation of a selection of B-PAS in a Norwegian bachelor’s level nursing programme. We contribute to the discussion of what skills should be taught, how the skills are taught over time, and to the call for close collaboration between the university faculty and preceptors in clinical rotation. The main findings are that despite reducing the comprehensive PAS in the curriculum and focusing on a set of B-PAS, utilisation of the skills in clinical practice is still limited. These findings are interesting in the light of research that argues for the benefits of reducing the range of skills that are taught in nursing education [3, 8, 13, 14, 30].

Although the nursing students in the study did not use the full range of B-PAS, the results show an overall increase in the use of the skills over the 3-year nursing education. The median scores as seen in Table 2 indicate that the nursing students in general practice more of every skill throughout their education. B-PAS that include assessing vital signs are frequently used in both the second and third year. Assessing respiratory rate and assessing SpO_2_ (which is also part of the vital signs assessment) increased by more than 1.0 in median score from the first to third year. This is a positive progression regarding the use of EWS and the quick-SOFA criteria (early identification of sepsis) which is similar to results from other studies [26, 32]. Assessing vital signs is an important skill because research shows that changes in vital signs (for example, respiration rate, pulse, and blood pressure) are predictors for clinical deterioration [12, 25]. Osborne et al. [26] stress that it is important to take into account factors such as comorbidity, advanced aging and frailty, polypharmacy, and fluid-electrolyte balance when assessing vital signs. Assessing vital signs is a part of B-PAS; it is important to combine the skills applied with clinical reasoning based on human biosciences in all clinical rotations throughout nursing education. By discussing and interpreting the collected data, nursing students will be able to articulate their decision-making processes and nursing practice to a greater extent [7, 18]. By understanding this, the preceptors can be key persons in supporting the nursing students to articulate knowledge from their studies on human biosciences, and in influencing the development of clinical judgement.

One might assume that practicing the same set of B-PAS every year would facilitate a greater progression between the first-year and the third-year students. However, it is not enough to practice B-PAS only in the university skills lab, and the nursing students themselves highlight the importance of practicing these skills in direct patient care. If the students do not practice B-PAS in clinical rotation, they are not likely to gain the confidence to practice them later. As shown in earlier studies, knowledge and competence without confidence will likely lead to PAS not being applied in clinical work [11, 21].

All students reported a median score below 4 in all the skills that included *percussion* and *auscultation*, meaning that they used these skills to a small extent or did not know how to perform them. These findings are similar to other study results [3, 13, 14, 30].

The varied utilisation of B-PAS indicates that contextual factors might have a greater impact on usage of B-PAS than anticipated, which is supported by the results from the focus group interviews. The fact that the nursing students do not have the confidence or opportunity to apply all of the B-PAS does not necessarily indicate that changes must be made regarding the skills taught in the education programme. As pointed out by others, this might rather indicate that the work environment and preceptor’s choice of methods have a greater and more complex influence on the processes related to the utilisation of B-PAS [8, 9, 23].

The nursing students practice B-PAS in a work environment that does not yet have a culture for, or supports, these types of nursing skills. The focus group interviews highlighted these perspectives, suggesting that the nursing students have to rely on their own motivation to develop and apply B-PAS rather than on guidance from their preceptors. This can also indicate that the focus for the students is on proficiencies, not on articulating knowledge, discussing cues and findings, or developing clinical reasoning skills. The challenging work environment for the nursing students in this study might have influenced the anticipated progression in the use of B-PAS.

Through the focus group interviews, the students explained that negative attitudes or critical comments from their preceptor or other healthcare workers could hinder their use of B-PAS and thus present a challenge to autonomous nursing work. Good examples of this are B-PAS that the students viewed as technically difficult, such as *percussion* and *auscultation* skills, as well as the skills related to *neurological assessment*. In practice, the students cannot rely on the preceptor’s guidance because the preceptor does not have B-PAS in their nursing education or as part of their current repertoire. Values and culture can be a barrier for successful implementation and can be a source of tension or conflict between those nurses who do not have these skills and, as in our study, the nursing students who need to learn these skills [8, 23]. The tension in the Norwegian context might explain why the nursing students in this study are reluctant to use B-PAS in their clinical rotations. When asked, more than half of the students believed that greater collaboration with preceptors would increase their learning outcomes related to the use of B-PAS. This also emphasises that the university must work more strategically with the preceptors in clinical practice. This includes offering clinical courses in B-PAS for the preceptors to increase their understanding of what the students learn in undergraduate education, as well as how to facilitate learning processes related to PAS. By organising training courses for preceptors, they could better support the students in utilising B-PAS when in clinical rotation.

The expectation is that with time, the impact of inhibitory factors in the work environment in the Norwegian context will be reduced as B-PAS become better known, and there are more examples of how they can positively influence nursing practice. Despite the reduced number of PAS in the curriculum, the results presented here are still similar to those of international studies where a small set of skills was used. This illustrates how important it is to discuss what skills should be taught in a bachelor’s programme, how the skills are taught, and the need for close collaboration with preceptors in clinical rotation [9].

### Limitations and strengths of the study

This cohort study used a single campus site for data collection in the Norwegian context. A strength of the study is the overall good response rate to the questionnaire. Due to the recent implementation of B-PAS, it was necessary to elicit student experiences with these curricular elements. This is a first endeavour to evaluate the programme and identify possible needs for change and modification before further deployment at other campuses. One limitation of the study design was not having all three cohorts, but we deemed it unfeasible to wait until we had a baseline for three different cohorts. Despite the limitations, our results show a strong similarity to the findings from other studies [3, 13, 14, 30]. We included a smaller group of participants in the focus group interviews, and the results must be interpreted with some caution. However, as the first study in Norway, the focus group interviews contributed to a better understanding of the challenges nursing students experienced when applying these skills in clinical rotation, especially in terms of factors that might hinder or facilitate further learning.

## Conclusion

Despite reduced PAS being taught in the curriculum, there is still a lack of use of B-PAS in clinical rotations. Even though B-PAS are not fully used, the results do indicate that they are increasingly used in clinical rotation throughout the 3-year nursing programme. This paper highlights that further studies are needed, both in a Norwegian and international context, in order to explore how the learning environment and preceptors’ attitudes influence the nursing students’ utilisation of B-PAS in combination with vital sign assessment. By discussing possible findings, the preceptors can promote nursing students’ articulation of knowledge from their studies on human biosciences, and they can influence the development of clinical judgement. It is important to assess whether the selected B-PAS are appropriate to teach in the 3-year undergraduate nursing curricula, and to continue to map the use of these skills in clinical practice. The study indicates that the nursing students are selective in what skills they apply in daily practice, but it remains essential that they apply B-PAS in a skills lab (a safe environment) and during clinical rotation in different contexts. This can positively change their ability to utilise their skills, and to develop competence and confidence as well as clinical judgement. It is important to explore different types of on-campus learning methods related to PAS. In addition, it will be important to further explore how clinical virtual simulation and reflection can stimulate critical thinking related to the development of clinical judgement [27].

## Data Availability

The quantitative datasets used and analysed during the current study are available from the corresponding author on reasonable request.
